# Assessment of common somatic mutations of *EGFR*, *KRAS*, *BRAF*, *NRAS* in pulmonary non-small cell carcinoma using iPLEX^®^ HS, a new highly sensitive assay for the MassARRAY^®^ System

**DOI:** 10.1371/journal.pone.0183715

**Published:** 2017-09-19

**Authors:** Bobbie C. Sutton, Ryan T. Birse, Kevin Maggert, Tammy Ray, Jessica Hobbs, Amobi Ezenekwe, Jason Kazmierczak, Michael Mosko, Joan Kish, Andrew Bullock, Zonggao Shi, M. Sharon Stack, Darryl Irwin

**Affiliations:** 1 Pathology Department, South Bend Medical Foundation, South Bend, IN, United States of America; 2 Agena Bioscience, San Diego, CA, United States of America; 3 Harper Cancer Research Institute, University of Notre Dame, Notre Dame, IN, United States of America; Universita degli Studi di Napoli Federico II, ITALY

## Abstract

Increased early detection and personalized therapy for lung cancer have coincided with greater use of minimally invasive sampling techniques such as endobronchial ultrasound-guided biopsy (EBUS), endoscopic ultrasound-guided biopsy (EUS), and navigational biopsy, as well as thin needle core biopsies. As many lung cancer patients have late stage disease and other comorbidities that make open surgical procedures hazardous, the least invasive biopsy technique with the highest potential specimen yield is now the preferred first diagnostic study. However, use of these less invasive procedures generates significant analytical challenges for the laboratory, such as a requirement for robust detection of low level somatic mutations, particularly when the starting sample is very small or demonstrates few intact tumor cells. In this study, we assessed 179 clinical cases of non-small cell lung carcinoma (NSCLC) that had been previously tested for *EGFR*, *KRAS*, *NRAS*, *and BRAF* mutations using a novel multiplexed analytic approach that reduces wild-type signal and allows for detection of low mutation load approaching 1%, iPLEX^®^ HS panel for the MassARRAY^®^ System (Agena Bioscience, San Diego, CA). This highly sensitive system identified approximately 10% more *KRAS*, *NRAS*, *EGFR* and *BRAF* mutations than were detected by the original test platform, which had a sensitivity range of 5–10% variant allele frequency (VAF).

## Introduction

In 2012, an estimated 14.1 million new cancer cases were diagnosed worldwide, and this number is predicted to rise over the coming years [1]. Lung cancer is the most frequent cancer worldwide, with nearly 1.83 million new cases of lung cancer estimated to have been diagnosed globally in 2012. Lung cancer is also the leading cause of cancer death in the United States, where an estimated 222,500 new cases will be diagnosed in 2017, with 155,870 deaths due to disease [2].

Traditionally, lung cancer survival rates tend to vary markedly depending on the stage at time of diagnosis. Unfortunately, many lung cancers are identified in the later stages of disease, translating to lower survival rates [3, 4]. These findings suggest that treatment can be significantly improved by detecting lung cancer tumors while they are smaller and more locally defined [3, 4]. However, more frequent biopsy of earlier, smaller tumors and increasing use of innovative, minimally invasive biopsy technologies have resulted in smaller samples with less tumor tissue available for analysis.

There has been a significant expansion of targeted therapies for NSCLC that have been shown to be effective in patients with specific genetic alterations expressed in tissue from their lung tumor, such as selected mutations in exons 18,19, 20 and 21 of *EGFR*. However, as knowledge of the histologic tumor type drives molecular studies, often a limited tumor sample becomes even smaller after diagnostic immunohistochemical stains are performed to distinguish pulmonary adenocarcinoma (PA) from squamous cell carcinoma, or a metastatic tumor from another organ. Any remaining tumor tissue must then be shared between multiple molecular genetic assays. With requirements to do more with less, it has been challenging for laboratories to establish an effective strategy for triaging specimens for molecular analysis of lung cancer. Additionally, multiple test platforms are in use in laboratories today to detect such mutations, many with an assay sensitivity ranging between 5 and greater than 20% variant allele frequency (VAF). Some of these test systems, such as Sanger sequencing [5,6], will miss mutations that are present at a low VAF, or if the tumor cellularity is less than 25–40%, both possibilities that are more likely in a limited tissue sample. In this clinical research study, we assessed 179 clinical cases of NSCLC previously tested for *EGFR*, *KRAS*, *NRAS*, *and BRAF* mutations using a novel multiplexed analytic approach that reduces wild-type signal and allows for detection of low mutation load approaching 1%, iPLEX^®^ HS panel for the MassARRAY^®^ System (Agena Bioscience, San Diego, CA).

## Material and methods

### Lung tumor samples

Archived frozen deoxyribonucleic acid (DNA) samples were searched for lung tumor cases previously tested for *EGFR*, *KRAS*, *NRAS and BRAF* mutations using the OncoFOCUS™ Panel v2.0 or v3.0 on the MassARRAY^®^ System (Agena Bioscience, San Diego, CA, USA). Specimens were de-identified prior to entry into the study. DNA originated from formalin-fixed, paraffin-embedded (FFPE) human clinical PA tissue samples. Of 184 lung tumor samples, 2 were excluded from the study due to insufficient original sample to complete testing, 2 were excluded due to iPLEX^®^ HS test failure, and was one excluded because of poor quality DNA in the residual specimen. Of the 179 remaining cases, only 38 were from larger excision specimens with plentiful tumor. Most specimens (129) were small biopsies, often thin caliber needle cores, and 12 were cytology cell blocks. All histologic diagnoses were confirmed by a pathologist. Most of the tumors were pure adenocarcinoma. Six cases demonstrated mixed squamous or neuroendocrine differentiation, one showed a sarcomatoid tumor component, and 11 were NSCLC not otherwise specified based on the available sample. Minimum tumor cellularity for analysis was set at 20%. DNA was extracted using the QIAamp DNA FFPE Tissue Kit (Qiagen, Boston, MA). Prior to repeat testing, all specimens were assessed for DNA integrity using the iPLEX^®^ Pro Sample ID Panel, and all specimens with adequate amplifiable DNA were then interrogated with a new, highly sensitive single polymerase chain reaction (PCR) iPLEX^®^ HS panel that includes more than 76 common mutations [7] in *BRAF*, *EGFR*, *KRAS*, *NRAS*, *and PIK3CA*; both panels were run on the MassARRAY^®^ System. During the development of the iPLEX^®^ HS chemistry a wide range of input DNA was tested (S1 Fig) and 5-10ng was found to be the optimal starting concentration.

### SNP genotyping

Genotyping of SNPs was performed using the iPLEX^®^ HS panel on the MassARRAY^®^ System (Agena Bioscience, San Diego, CA, USA), which employs matrix-assisted laser desorption/ionization time-of-flight mass spectrometry for amplicon detection (MALDI-TOF-MS; SpectroACQUIRE, Agena Bioscience). Primers designed for PCR (polymerase chain reaction) amplification of specific mutations in *BRAF*, *EGFR*, *KRAS*, *NRAS*, and *PIK3CA*, and extension reactions were prepared using the MassARRAY^®^ Assay Design Version 3.1 software (Agena Bioscience, San Diego, CA, USA). PCR reactions contained: *Taq* DNA polymerase (Agena Bioscience), genomic DNA (5–10 ng), PCR primers, and dNTP. Following PCR (45 cycles), the remaining dNTPs were removed by the addition of alkaline phosphatase (Agena Bioscience), after which the plates were incubated at 37°C for 40 min. (as previously described in [8]).

**Fig 1:**
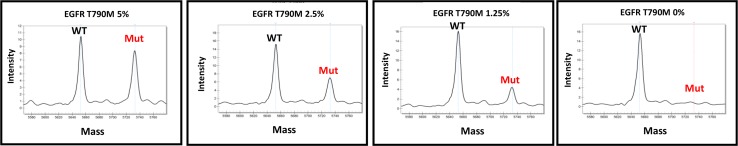
EGFR T790M dilution series. Example of a dilution series for detection of EGFR-T790M mutation (Horizon Discovery-Boston, Cambridge MA), showing spectral peaks of mutation and WT from 5% mutation VAF down to 0%.

**Fig 2:**
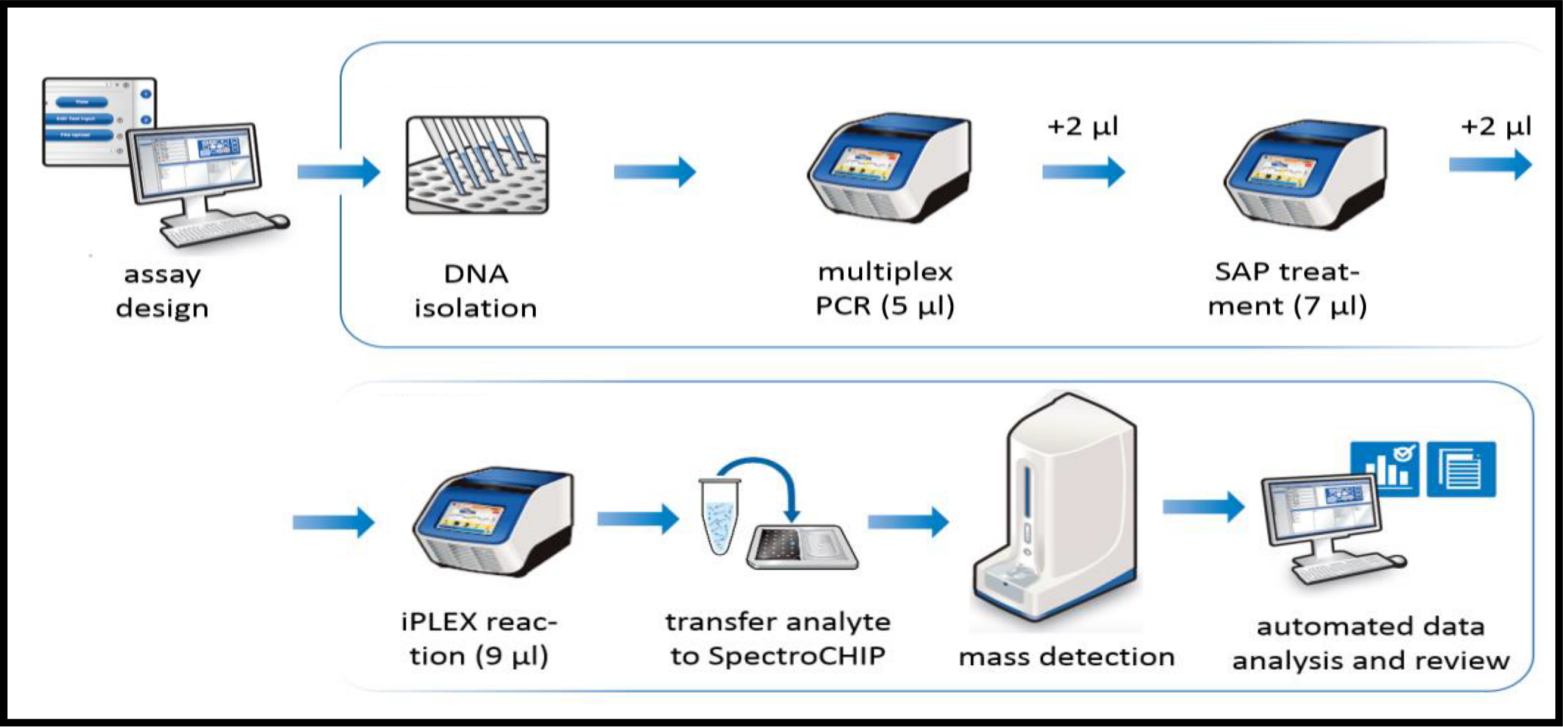
Workflow schemtic. Schemtic workflow for somatic mutation detection using iPLEX^®^ chemistry and the MassARRAY^®^ System. Total turn around time from DNA to data is less than 8 hours.

iPLEX^®^ HS chemistry is a wild-type (WT) terminator-depleted chemistry designed to reduce the wild-type extension terminator signal in a DNA specimen. This allows for quantification of a mutation down to a very low variant allele frequency (VAF) as the analytical window is not dominated by the wild-type allele (S2 Fig). Following the PCR reaction, SAP addition, and iPLEX HS^®^ extension reaction, the samples were desalted by resin treatment for 15 min, spotted onto SpectroCHIP^®^ Arrays (Agena Bioscience, San Diego, CA), analyzed by mass spectrometer, and ultimately interpreted on SpectroTYPER v4.0 software (Agena Bioscience, San Diego, CA). A mutation signal produced using iPLEX^®^ HS chemistry can be reliably detected by the MassARRAY^®^ System at about 1% VAF (see Fig 1). iPLEX^®^ HS assays can be performed within 8 hrs from DNA to reportable result, which is amenable to requirements for turnaround time currently in place for lung cancer tumor mutation analysis in clinical laboratories [9]. For an overview of these experimental processes, see Fig 2.

### Digital droplet PCR

The QX200 Droplet Digital PCR System reaction was performed in biological triplicates per manufacturer’s instructions for use [10]. The ddPCR contained 10 uL of the various genomic mixes, 12.5 uL ddPCR super mix, and 2.5 uL primer/probe mix. Primers and TaqMan probes for *KRAS* (G12C, G13D, G12D, G13C, G12V), *EGFR* (L858R), *NRAS* (G13R), and *BRAF* (V600E) mutations were purchased from existing mutation assays from Bio-Rad (Hercules, CA). PCR components were separated into individual reaction vessels using the QX100 Droplet Generator (Bio-Rad, Hercules, CA). The droplet generation process combines 70 uL of droplet generation oil with 20 uL of the ddPCR. This process was performed in a cartridge with a cartridge holder and droplet generation gasket. Subsequent to droplet formation, 40 uL of the formed droplet reaction was transferred from the cartridge to a 96-well PCR plate. Amplification parameters were as follows: 95°C for 10 minutes, followed by 40 cycles of 94°C for 30 seconds, and 55°C for 1 minute. Cycling was followed by 98°C incubation for 10 minutes. Annealing temperature was experimentally defined [10]. After the reaction, t he droplets were read using the Droplet Reader (Bio-Rad), and QuantaSoft software version 1.4.0.99 (Bio-Rad) converted the data into concentrations using Poisson distribution statistical analysis.

## Results

### High sensitivity iPLEX^®^ HS somatic mutation detection

**Fig 3:**
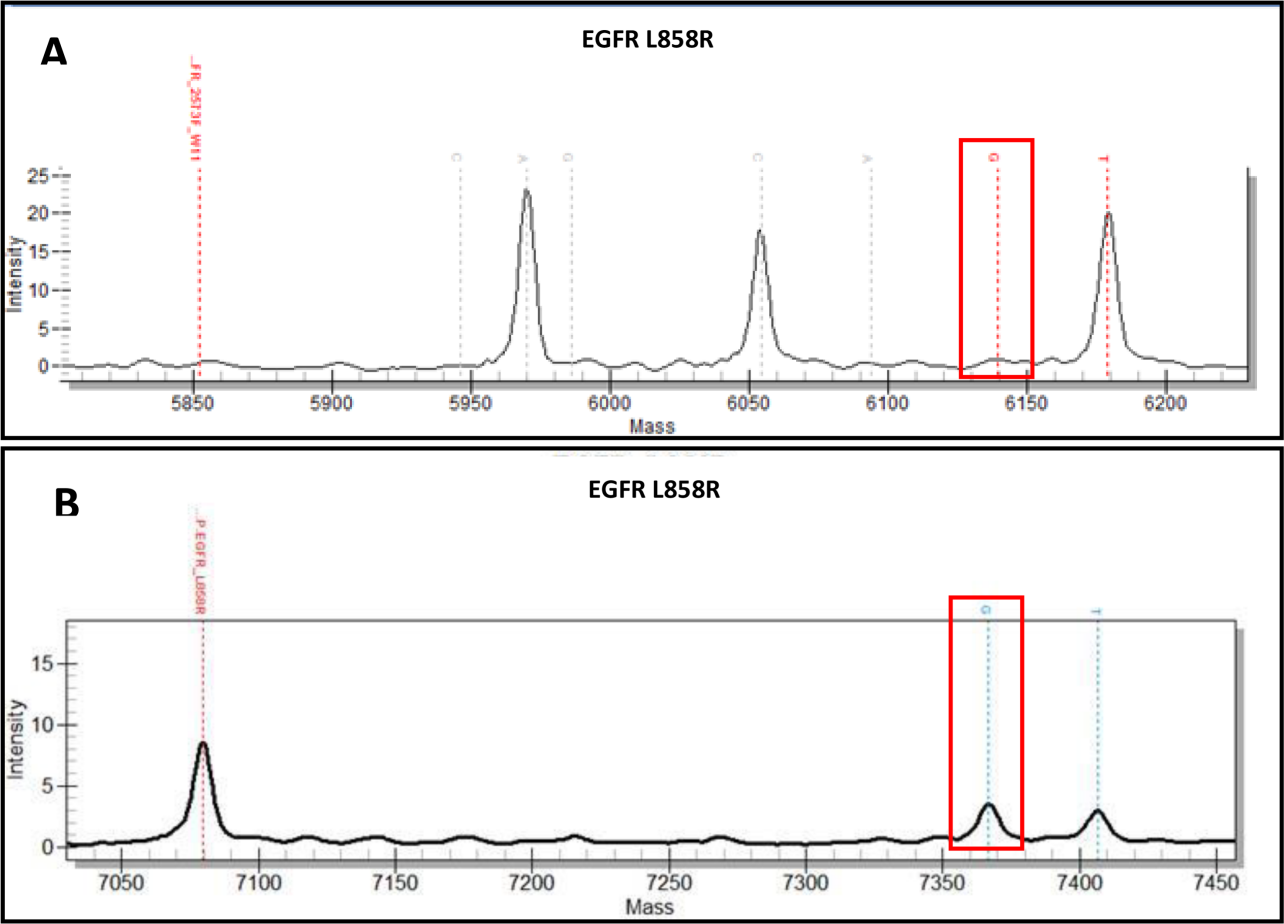
5% LOD vs 1% LOD spectrums. An example spectral data comparison of an EGFR p.L858R mutation (red box) from the same sample run on both the OncoFOCUS™ Panel (top) and the iPLEX^®^ HS panel (bottom).

In 179 samples, mutations in *KRAS* (n = 55; 55/179 = 30.7%), *BRAF* (n = 8; 8/179 = 4.5%), *EGFR* (n = 19; 19/179 = 10.6%), and *NRAS* (n = 3; 3/179 = 1.7%) were detected using iPLEX^®^ HS chemistry, for a total of 85 mutations observed. This correlates well with other reports of genetic analysis of pulmonary adenocarcinoma, where mutation frequencies range from 10–21% of tumors with mutated *EGFR*, 25–33% of tumors with mutated *KRAS*, and 2–10% with mutated *BRAF*, while *NRAS* mutations are rare, depending on the patient population studied [3,11,12,13]. When compared to previous results from the OncoFOCUS^TM^ Panel, which has a sensitivity of approximately 5–10% VAF, this represents an additional 17 previously undetected mutations (17/179, or 9.5% more mutations detected). The mass spectra from the original runs were reviewed, and several mutations were considered suspect but unconfirmed due to weak spectral peaks or low confidence calls by the system software (See Table 1, S1 Table). However, some of the new mutations could not be identified in the original data run (See Table 1, S1 Table). Therefore, by improving the level of detection from 5–10% down to 1–5%, we confirmed 8 mutations that were previously considered probable in the original data, including two *EGFR* L858R mutations (Table 1). An example of spectral data comparison from the same sample run on both OncoFOCUS™ and iPLEX^®^ HS panels is shown in Fig 3. In two cases a second previously undetected mutation was identified, while an additional 6 new *KRAS* mutations, 2 *NRAS*, 1 *BRAF*, and 1 *EGFR* mutation were identified (See Table 1). While 3 of these cases originated from larger excision specimens, the most common sample type where an additional mutation was identified was a needle core biopsy, and 2 cases originated from cytology cell blocks (See Table 1). Additionally, 4/179, or 2% of cases demonstrated a mutation in *PIK3CA*, which was not interrogated by the OncoFOCUS^TM^ Panel. This mutation frequency is similar to other reports [11,12]. Sensitivity of the iPLEX^®^ HS panel was confirmed by testing dilution series of specimens with known mutant allele vs. WT copy number prepared using commercial DNA standards (Horizon Discovery-Boston, Cambridge MA); see Fig 1.

**Table 1:** This table lists the the additional mutations identified by the iPLEX^®^ HS panel, and how they were evaluated. ND: None detected (includes only mutations common to both panels).

Case	iPLEX^®^HS Mutant Call	iPLEX^®^HS 2nd Mutation	Original result	Specimen Type	Comments
TMF-19	*EGFR* L858R	ND	ND	Core biopsy	On original spectrum mutation was identified by system software and small mutant peak was present, but not significantly above baseline.
TMF-22	*KRAS* G12C	ND	ND	Core biopsy	On original spectrum mutation was identified by system software and small mutant peak was present, but not significantly above baseline.
TMF-28	*BRAF* V600E	*KRAS* G12C	*KRAS* G12C	Core biopsy	*BRAF* V600E not identified by software in original run. **Mutation confirmed by ddPCR assay.**
TMF-29	*KRAS* G13D	ND	ND	Core biopsy	On original spectrum mutation was identified by system software and small mutant peak was present, but not significantly above baseline.
TMF-37	*BRAF* V600E	*NRAS* G13R	*BRAF* V600E	Core biopsy	*NRAS* G13R not identified by software in original run. **Mutation confirmed by ddPCR assay.**
TMF-63	*KRAS* G12C	ND	ND	Cytology cell block	*KRAS* G12C not identified by software in original run. **Mutation confirmed by ddPCR assay.**
TMF-69	*KRAS* G13D	ND	ND	Core biopsy	*KRAS* G13D not identified by software in original run. **Mutation confirmed by ddPCR assay.**
TMF-76	*BRAF* V600E	ND	ND	Core biopsy	On original spectrum no call by software, but two composite PCR reactions show very weak mutant allele peaks.
TMF-80	*KRAS* G12D	NRAS G12D	ND	Core biopsy	*KRAS* G12D not identified by software in original run. **Mutation confirmed by ddPCR assay.** NRAS G12D called by software but could not make confidant call.
TMF-104	*KRAS* G12V	ND	ND	Core biopsy	*KRAS* G12V not identified by software in original run. **Mutation confirmed by ddPCR assay.**
TMF-135	*BRAF* V600E	ND	ND	Core biopsy	On original spectrum no call by software, but two composite reactions show very weak mutant allele peaks.
TMF-136	*EGFR* L858R	ND	ND	Excision	On original spectrum mutation was identified by system software and a small, weak mutant peak was present. Mutation did not confirm with secondary PCR.
TMF-141	*BRAF* V600E	ND	ND	Cytology cell block	On original spectrum no call by software, but two composite reactions show very weak mutant allele peaks.
TMF-144	*NRAS* G13R	ND	ND	Core biopsy	*NRAS* G13R not identified by software in original run. **Mutation confirmed by ddPCR assay.**
TMF-151	*KRAS* G12D	ND	ND	Excision	*KRAS* G12D not identified by software in original run. **Mutation confirmed by ddPCR assay.**
TMF-173	*BRAF* V600E	ND	ND	Excision	No software call *BRAF* V600E. **Mutation confirmed by ddPCR assay.**
TMF-182	*EGFR* L858R	ND	ND	Core biopsy	On original spectrum mutation was identified by system software and a small mutant peak was present, but not significantly above baseline. **Mutation confirmed by ddPCR assay.**

### Digital droplet verification

**Fig 4:**
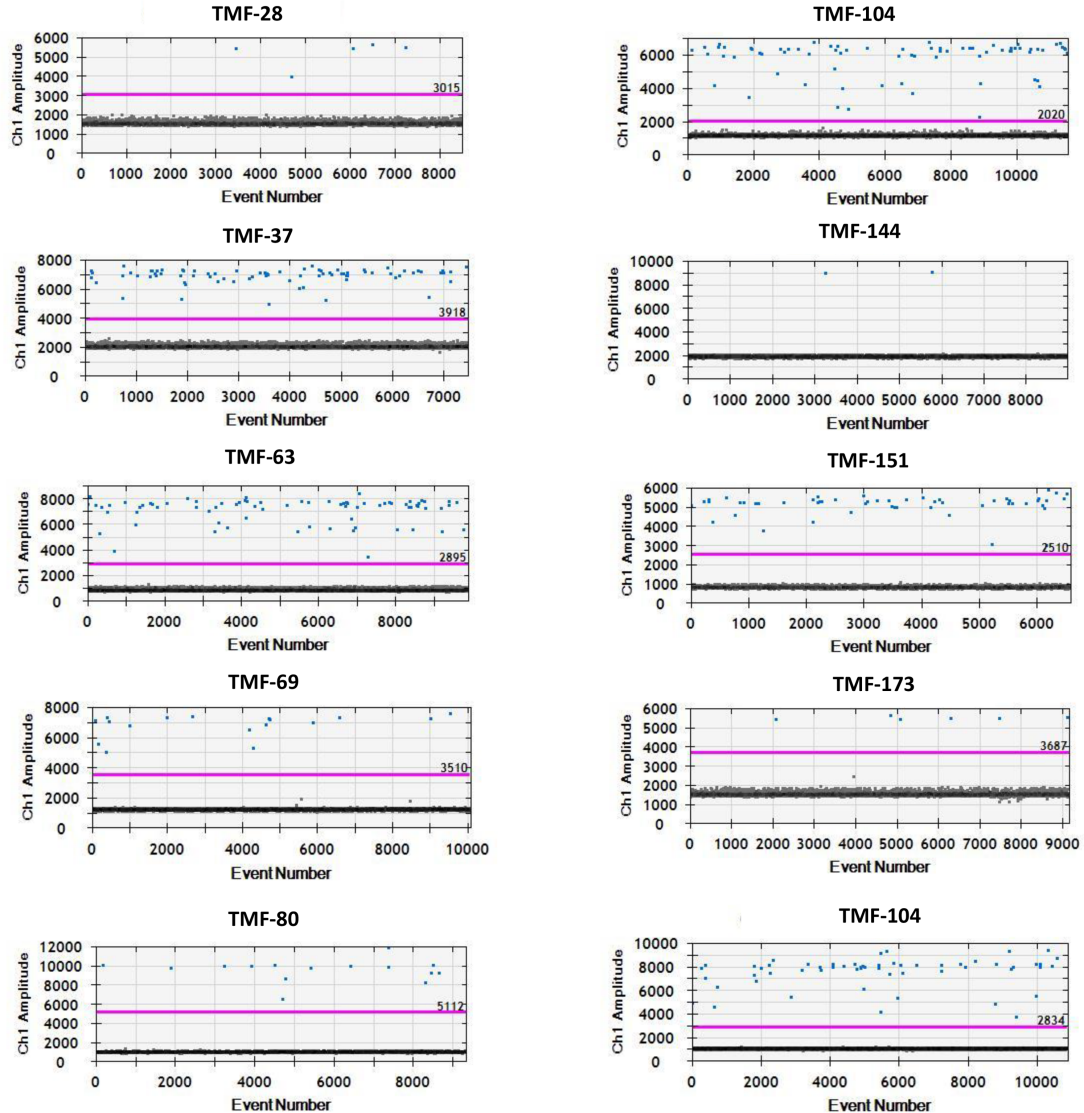
ddPCR graphs. ddPCR graphs of mutant (blue pixels) vs blank (black pixels) events with a detection threshold (purple line). Patient samples; TMF-28 (BRAF-V600E), TMF-37 (BRAF-V600E), TMF-63 (KRAS-G12C), TMF-69 (KRAS-G13D), TMF-80 (KRAS-G12D), TMF-104 (KRAS-G12V), TMF-144 (NRAS-G13R), TNF-173 (BRAF-V600E), TMF-182 (EGFR-L858R).

Digital droplet PCR (ddPCR) was used to verify the new mutations which were identified using the iPLEX^®^ HS panel but which were previously unconfirmed or not detected by OncoFOCUS™ Panel analysis. Using the QX200 Droplet Digital PCR System we performed tests in biological triplicates per manufacturer’s instructions for use (9). Rare event detection assays were employed for both mutation and WT probes for *KRAS* (G12C, G13D, G12D, G13C, G12V), *EGFR* (L858R), *NRAS* (G13R), and *BRAF* (V600E) mutations (Bio-Rad, Hercules, CA) (Fig 4). As a positive control a dilution series of mutant allele vs. WT copy number was prepared using commercial DNA standards [*KRAS* (G12C, G13D, G12D, G13C, G12V), *EGFR* (L858R), and *BRAF* (V600E); (Horizon Discovery-Boston, Cambridge MA) (Fig 4, S1 Table). We positively identified and confirmed all 10 previously undetected mutations, thereby verifying the iPLEX^®^ HS detection chemistry (Fig 4, S1 Table).

## Discussion

Early detection and appropriate testing of lung cancer is of vital importance to improving patient outcomes. This consequently places a considerable burden on pathologists to ensure that specimens used for molecular testing meet the laboratory’s requirements for tumor content and quality. However, this can be challenging. Other groups have shown that tissue specimens with low tumor cellularity may lead to false-negative mutation results, particularly if an assay has a relatively low analytic sensitivity, such as Sanger sequencing [5,6]. This problem may be encountered with any small, limited tumor tissue sample. Dudley et al [14] reported that tumor cell depletion by neoadjuvant therapy for rectal adenocarcinoma could impede detection of *KRAS* mutations. Chen et al [15] demonstrated that detection of *BRAF* mutations in lymph nodes involved with metastatic malignant melanoma was particularly challenging when there was a low tumor cell burden, such as with small subcapsular metastatic deposits or infiltrative tumor cells in a background of small lymphocytes. The use of a highly sensitive molecular assay is critical for accurate assessment of such specimens. In this clinical research study, we demonstrate similar findings for mutation analysis in NSCLC. By increasing the assay sensitivity from 5–10% VAF detection to 1%, the iPLEX^®^ HS panel and MassARRAY^®^ System resulted in the identification of 17 (17/179, or 9.5%) previously undetected *KRAS*, *NRAS*, *BRAF*, and *EGFR* mutations. Furthermore, with an 8 hour turnaround time and low per sample cost, we can perform targeted screens on more samples with a higher level of detection and thereby potentially discover mutations at an earlier stage.

We also show that low VAF mutations can be reliably detected in small tissue samples, such as needle core biopsies and cytology cell block specimens, which are increasingly seen in clinical molecular pathology laboratories.

## Supporting information

S1 Table Oncofocus vs iPLEX^®^ HS sample comparison. Table of all the results from the samples tested with the OncoFOCUS™ and iPLEX^®^ HS chemistries. NA, not applicable.
(DOCX)

S2 Table Mutants validated with ddPCR. Table of mutants validated with ddPCR (Droplet Digital PCR System. Hercules, CA). Patient samples; TMF-28 (BRAF_V600E), TMF-37 (BRAF_V600E), TMF-63 (KRAS_G12C), TMF-69 (KRAS_G13D), TMF-80 (KRAS_G12D), TMF-104 (KRAS_G12V), TMF-144 (NRAS_G13R), TNF-173 (BRAF_V600E), TMF-182 (EGFR_L858R). Copy number and the microliter amount added to the PCR reaction. All samples were tested with a minimum of 4 biological replicates. Positive calls were the number of mutant calls out of the total number of biological replicates. Allelic frequency is presented as an average of the successful runs. Comments referring to “Present in OncoFocus spectrum but not significantly above baseline” is meant to indicate that there was a weak call which when present in the iPLEX^®^ HS chemistry was sufficient evidence that the mutation was present in the old spectra. Please refer to Supplements S2 Table for the full list of patient samples orthogonally validated using ddPCR.
(DOCX)

S3 Table iPLEX^®^ HS mutation list. Full list of all mutations covered in the iPLEX^®^ HS panel.
(DOCX)

S1 Fig Dose response for input DNA for mutation PI3KCA E542K. Mutation PI3KCA E542K was used a representative of all mutations tested. Graphs A-D are minor variant detection of PI3KCA E542K at input DNA concentrations ranging from 1ng, 5ng, 10ng and 20 ng. An all pairs Tukey-Kramer test was performed on all comparisons at a p value = 0.05.
(TIF)

S2 Fig iPLEX^®^ Pro vs iPLEX^®^ HS. Comparison of iPLEX^®^ Pro vs iPLEX^®^ HS level of detection of minor variants. Data was analyzed using signal to noise ratio to identify prominent differences in peaks.
(TIF)

## References

[1] World Cancer Report 2014.

[2] Siegel RL, Miller KD, Jemal A. Cancer Statistics, 2016. CA Cancer J Clin 2016; 66:7–30. doi: 10.3322/caac.21332 26742998 10.3322/caac.21332

[3] Henschke CI, Yankelevitz DF. CT screening for lung cancer: update 2007. Oncologist 2008 13(1): 65–78. doi: 10.1634/theoncologist.2007-0153 18245013 10.1634/theoncologist.2007-0153

[4] National Comprehensive Cancer Network Clinical Practice Guidelines in Oncology. Non-Small Cell Lung Carcinoma v4.2017.

[5] Tol J, Dijkstra JR, Vink-Börger ME, Nagtegaal ID, Punt CJ, Van Krieken JH et al, High sensitivity of both sequencing and real-time PCR analysis of *KRAS* mutations in colorectal cancer tissue. J Cell Mol Med; 14(8), 2010 2122–2131. doi: 10.1111/j.1582-4934.2009.00788.x 19453520 10.1111/j.1582-4934.2009.00788.x PMC3823003

[6] Angulo B, García-García E, Martínez R, Suárez-Gauthier A, Conde E, Hidalgo M, et al, A commercial Real-Time PCR Kit Provides Greater Sensitivity than Direct Sequencing to Detect *KRAS* Mutations. J Mol Diag 2010 12(3): 292–299. 10.2353/jmoldx.2010.090139 PMC2860464 20203003

[7] http://agenabio.com/wp-content/uploads/2017/03/Agena-Bioscience-iPLEX-HS-Lung-Panel-Mutation-List_WEB_0317.pdf.

[8] http://agenabio.com/wp-content/uploads/2016/02/51-20061R3.0_iPLEX_Chemistry_App_Note_0216_WEB.pdf.

[9] Lindeman NI, Cagle PT, Beasley MB, Chitale DA, Dacic S, Giaccone G. Molecular Testing Guideline for Selection of Lung Cancer Patients for *EGFR* and *ALK* Tyrosine Kinase Inhibitors. Guideline from the College of American Pathologists, International Association for the Study of Lung Cancer, and the Association for Molecular Pathology. J Mol Diag 2013 15; 415–453. 10.1016/j.jmoldx.2013.03.001 23562183

[10] Bio-Rad Technical note: Detection of Rare Mutant Alleles within a Background of Wild-Type Sequences Using the QX100 Droplet Digital PCR System. Hercules, CA

[11] Kris MG, Johnson BE, Berry LD, Kwiatkowski DJ, Iafrate AJ, Wistuba II, et al, Using Multiplexed Assays of Oncogenic Drivers in Lung Cancers to select Targeted Drugs. JAMA. 2014; 311(19): 1998–2006. doi: 10.1001/jama.2014.3741 24846037 10.1001/jama.2014.3741 PMC4163053

[12] Gainor JF, Varghese AM, Ignatius Ou SH, Kabraji S, Awad MM, Katayama R, et al 2013. *ALK* rearrangements Are Mutually Exclusive with Mutations in *EGFR* or *KRAS*: An analysis of 1683 patients with Non-Small Cell Lung Cancer. Clin Cancer Res. 2013 19(15): doi: 10.1158/1078-0432.CCR-13-0318 23729361 10.1158/1078-0432.CCR-13-0318 PMC3874127

[13] Comprehensive molecular profiling of lung adenocarcinoma. The Cancer Genome Atlas Research Network. Nature 2014 Vol 511; 7. 10.1038/nature13385 PMC4231481 25079552

[14] Dudley J, Tseng LH, Rooper L, Harris M, Haley L, Chen G, et al Challenges Posed to Pathologists in the Detection of KRAS Mutations in Colorectal Cancers. Arch Pathol Lab Med 2015 139; 211–218. doi: 10.5858/arpa.2013-0649-OA 25611103 10.5858/arpa.2013-0649-OA

[15] Chen G, Dudley J, Tseng LH, Smith K, Gurda GT, Gocke CD et al Lymph node metastases of melanoma: challenges for BRAF mutation detection. Human Pathol 2014 46, 113–119. 25456393 10.1016/j.humpath.2014.09.014

