# Supplementary material for: Assessment of common somatic mutations of EGFR, KRAS, BRAF, NRAS in pulmonary non-small cell carcinoma using iPLEX® HS, a new highly sensitive assay for the MassARRAY® System
Source: PLoS One. 2017 Sep 19;12(9):e0183715. doi: 10.1371/journal.pone.0183715 (PMC5604939; doi:10.1371/journal.pone.0183715)
Supplement: S1 Table — Table of all the results from the samples tested with the OncoFOCUS™ and iPLEX® HS chemistries. NA, not applicable. (DOCX) [file pone.0183715.s001.docx]

**Supplemental Information**

| **Sample ID** | **iPlex Pro OncoFocus (5-10% LOD) Detected Mutation/s** | **iPlexHS (1% LOD) Detected Mutation/s** | **Confirmation** | **Specimen type** |
| --- | --- | --- | --- | --- |
| TMF-1 |  |  | NA | Small biopsy |
| TMF-2 | EGFR_L858R | EGFR_L858R | Confirmed | Excision |
| TMF-4 |  |  | NA | Small biopsy |
| TMF-5 | KRAS_G12A | KRAS_G12A | Confirmed | Excision |
| TMF-6 |  |  | NA | Excision |
| TMF-7 | KRAS G12D | KRAS_G12D | Confirmed | Small biopsy |
| TMF-8 |  |  | NA | Small biopsy |
| TMF-9 |  |  | NA | Small biopsy |
| TMF-10 |  |  | NA | Excision |
| TMF-11 |  |  | NA | Small biopsy |
| TMF-12 | KRAS_G12C | KRAS_G12C | Confirmed | Excision |
| TMF-13 |  |  | NA | Small biopsy |
| TMF-14 |  |  | NA | Cytology |
| TMF-15 |  |  | NA | Small biopsy |
| TMF-16 | BRAF_D594G |  | NA - BRAF_D594G not in iPlex HS panel | Excision |
| TMF-17 |  |  | NA | Small biopsy |
| TMF-18 |  |  | NA | Small biopsy |
| TMF-19 |  | EGFR_L858R | Present in OncoFocus spectrum but not significantly above baseline | Small biopsy |
| TMF-20 |  | EGFR_p.E746-A750del_c.2235-2249del15 | Present in OncoFocus spectrum but not significantly above baseline | Small biopsy |
| TMF-21 | KRAS G12V | KRAS_G12V | Confirmed | Small biopsy |
| TMF-22 |  | KRAS_G12C | Present in OncoFocus spectrum but not significantly above baseline | Small biopsy |
| TMF-23 |  |  | NA | Small biopsy |
| TMF-24 |  |  | NA | Excision |
| TMF-25 |  | KRAS_G12C | Present in OncoFocus spectrum but not significantly above baseline | Small biopsy |
| TMF-26 |  |  | NA | Small biopsy |
| TMF-27 | BRAF_V600E | BRAF_V600E | Confirmed | Small biopsy |
| TMF-28 | KRAS_G12C | BRAF_V600E, KRAS_G12C | Confirmed - ddPCR confirmed BRAF V600E | Small biopsy |
| TMF-29 |  | KRAS_G13D | Present in OncoFocus spectrum but not significantly above baseline | Small biopsy |
| TMF-30 | KRAS_G12A | KRAS_G12A | Confirmed | Small biopsy |
| TMF-31 |  |  | NA | Excision |
| TMF-32 | KRAS_G12D | KRAS_G12D | Confirmed | Cytology |
| TMF-33 |  |  | NA | Small biopsy |
| TMF-34 |  |  | NA | Excision |
| TMF-35 | KRAS_G12C | KRAS_G12C | Confirmed | Excision |
| TMF-37 | BRAF_V600E | BRAF_V600E, NRAS_G13R | Confirmed - ddPCR confirmed NRAS G13R | Small biopsy |
| TMF-38 |  |  | NA | Cytology |
| TMF-39 | KRAS_G12D | KRAS_G12D | Confirmed | Small biopsy |
| TMF-40 | EGFR_p.E746-A750del_c.2235-2249del15 | EGFR_p.E746-A750del_c.2235-2249del15 | Confirmed | Small biopsy |
| TMF-41 |  |  |  | Excision |
| TMF-42 | KRAS_G12C | KRAS_G12C | Confirmed | Small biopsy |
| TMF-43 | KRAS_G12C | KRAS_G12C | Confirmed | Small biopsy |
| TMF-44 | KRAS G12C | KRAS G12C | Confirmed | Excision |
| TMF-45 | KRAS_G12C | KRAS_G12C | Confirmed | Small biopsy |
| TMF-46 |  |  | Confirmed | Small biopsy |
| TMF-47 | KRAS_G12C | KRAS_G12C | Confirmed | Small biopsy |
| TMF-48 | KRAS_G12C | KRAS_G12C | Confirmed | Small biopsy |
| TMF-49 | KRAS_G12A | KRAS_G12A | Confirmed | Small biopsy |
| TMF-50 | EGFR_p.E746-A750del_c.2236-2250del15 | EGFR_p.E746-A750del_c.2236-2250del15 | Confirmed | Small biopsy |
| TMF-51 | KRAS_G12C | KRAS_G12C | Confirmed | Small biopsy |
| TMF-52 | KRAS_G12C | KRAS_G12C | Confirmed | Small biopsy |
| TMF-53 | BRAF_K601E |  | NA - BRAF_K601E not in iPlex HS panel | Small biopsy |
| TMF-54 |  |  | NA | Small biopsy |
| TMF-55 |  | PIK3CA E542K, E545K, NRAS A59D | NA - NRAS A59D, PIK3CA not in OncoFOCUS | Small biopsy |
| TMF-56 |  |  | NA | Excision |
| TMF-57 | KRAS_G12C | KRAS_G12C | Confirmed | Small biopsy |
| TMF-58 |  |  | NA | Excision |
| TMF-59 | BRAF_K601E |  | NA - BRAF_K601E not in iPlex HS panel | Excision |
| TMF-60 |  | PIK3CA E542K, E545K | NA - PIK3CA not in OncoFOCUS | Excision |
| TMF-61 | EGFR_p.L747-P753>S_c.2240-2257del18 | EGFR_p.L747-P753>S_c.2240-2257del18 | Confirmed | Excision |
| TMF-62 |  |  | NA | Excision |
| TMF-63 |  | KRAS_G12C | ddPCR confirmed KRAS G12C. | Cytology |
| TMF-64 | KRAS_G12C | KRAS_G12C | Confirmed | Small biopsy |
| TMF-65 | EGFR_p.E746-A750del_c.2235-2249del15 | EGFR_p.E746-A750del_c.2235-2249del15 | Confirmed | Small biopsy |
| TMF-66 |  |  | NA | Excision |
| TMF-67 |  |  | NA | Small biopsy |
| TMF-68 | KRAS_G12C | KRAS_G12C | Confirmed | Small biopsy |
| TMF-69 |  | KRAS_G13D | ddPCR confirmed KRAS G13D. | Small biopsy |
| TMF-70 | KRAS_G12V | KRAS_G12V | Confirmed | Small biopsy |
| TMF-71 |  |  | NA | Small biopsy |
| TMF-72 | KRAS_G12C | KRAS_G12C | Confirmed | Small biopsy |
| TMF-73 |  |  | NA | Small biopsy |
| TMF-74 |  |  | NA | Small biopsy |
| TMF-75 |  |  | NA | Small biopsy |
| TMF-76 |  | BRAF_V600E | Present in OncoFocus spectrum but not significantly above baseline | Small biopsy |
| TMF-77 |  |  | NA | Small biopsy |
| TMF-78 |  |  | NA | Small biopsy |
| TMF-79 | KRAS_G12D | KRAS_G12D | Confirmed | Small biopsy |
| TMF-80 |  | KRAS G12D and NRAS G12D/E | ddPCR confirmed KRAS G12D. | Small biopsy |
| TMF-81 |  |  | NA | Small biopsy |
| TMF-82 |  |  | NA | Small biopsy |
| TMF-83 |  |  | NA | Small biopsy |
| TMF-84 |  |  | NA | Small biopsy |
| TMF-85 |  |  | NA | Cytology |
| TMF-86 | KRAS_G12V | KRAS_G12V | Confirmed | Small biopsy |
| TMF-87 |  |  | NA | Small biopsy |
| TMF-88 | KRAS_G12C | KRAS_G12C | Confirmed | Small biopsy |
| TMF-89 |  |  | NA | Small biopsy |
| TMF-90 |  |  | NA | Small biopsy |
| TMF-91 |  |  | NA | Small biopsy |
| TMF-92 |  |  | NA | Small biopsy |
| TMF-93 |  |  | NA | Cytology |
| TMF-94 | KRAS_G12C | KRAS_G12C | Confirmed | Small biopsy |
| TMF-95 | KRAS_G12C | KRAS_G12C | Confirmed | Small biopsy |
| TMF-96 |  |  | NA | Excision |
| TMF-97 |  |  | NA | Small biopsy |
| TMF-98 |  |  | NA | Cytology |
| TMF-99 | KRAS_G12D | KRAS_G12D | Confirmed | Small biopsy |
| TMF-100 | KRAS_G12V | KRAS_G12V | Confirmed | Small biopsy |
| TMF-101 | BRAF_V600E | BRAF_V600E | Confirmed | Small biopsy |
| TMF-103 |  |  | NA | Excision |
| TMF-104 |  | KRAS_G12V | ddPCR confirmed KRAS G12V | Small biopsy |
| TMF-105 | KRAS_G12V | KRAS_G12V | Confirmed | Excision |
| TMF-106 | KRAS_G12A | KRAS_G12A | Confirmed | Small biopsy |
| TMF-107 |  |  | NA | Small biopsy |
| TMF-108 |  |  | NA | Small biopsy |
| TMF-109 |  |  | NA | Small biopsy |
| TMF-110 | KRAS_G12C | KRAS_G12C | Confirmed | Small biopsy |
| TMF-111 |  |  | NA | Small biopsy |
| TMF-112 | KRAS_G12D | KRAS_G12D | Confirmed | Small biopsy |
| TMF-113 |  |  | NA | Small biopsy |
| TMF-114 | KRAS_G12V | KRAS_G12V | Confirmed | Small biopsy |
| TMF-115 |  |  | NA | Small biopsy |
| TMF-116 | KRAS_G12C | KRAS_G12C | Confirmed | Small biopsy |
| TMF-117 |  |  | NA | Small biopsy |
| TMF-118 |  |  | NA | Excision |
| TMF-119 | EGFR_p.E746-A750del_c.2236-2250del15 | EGFR_p.E746-A750del_c.2236-2250del15 | Confirmed | Small biopsy |
| TMF-120 |  |  | NA | Small biopsy |
| TMF-121 |  |  | NA | Small biopsy |
| TMF-122 |  |  | NA | Small biopsy |
| TMF-123 |  |  | NA | Excision |
| TMF-124 |  |  | NA | Small biopsy |
| TMF-125 |  |  | NA | Excision |
| TMF-126 |  |  | NA | Small biopsy |
| TMF-127 | KRAS_G12C | KRAS_G12C | Confirmed | Small biopsy |
| TMF-128 |  |  | NA | Cytology |
| TMF-129 |  |  | NA | Small biopsy |
| TMF-130 |  |  | NA | Excision |
| TMF-132 | ?L858R (not confirmed by PCR) | EGFR_L858R | Confirmed | Excision |
| TMF-133 | KRAS_G12C | KRAS_G12C | Confirmed | Small biopsy |
| TMF-134 |  |  | NA | Small biopsy |
| TMF-135 |  | BRAF_V600E | Present in OncoFocus spectrum but not significantly above baseline | Small biopsy |
| TMF-136 |  | EGFR L858R | Present in OncoFocus spectrum but not significantly above baseline | Excision |
| TMF-137 | KRAS_G12A | KRAS_G12A | Confirmed | Excision |
| TMF-138 | EGFR_p.E746-A750del_c.2235-2249del15 | EGFR_p.E746-A750del_c.2235-2249del15 | Confirmed | Small biopsy |
| TMF-139 | KRAS_G12C | KRAS_G12C | Confirmed | Small biopsy |
| TMF-140 | KRAS_G12D | KRAS_G12D | Confirmed | Small biopsy |
| TMF-141 |  | BRAF V600E | Present in OncoFocus spectrum but not significantly above baseline | Cytology |
| TMF-142 | EGFR_L858R | EGFR_L858R | Confirmed | Small biopsy |
| TMF-143 |  |  | NA | Cytology |
| TMF-144 | (KRAS) A146T | NRAS_G13R | ddPCR confirmed NRAS G13R. KRAS A146T not in iPlexHS panel. | Small biopsy |
| TMF-145 | EGFR_L858R | EGFR_L858R | Confirmed | Cytology |
| TMF-146 |  |  | NA | Small biopsy |
| TMF-147 |  |  | NA | Small biopsy |
| TMF-148 |  |  | NA | Small biopsy |
| TMF-149 |  |  | NA | Small biopsy |
| TMF-150 |  |  | NA | Small biopsy |
| TMF-151 |  | KRAS_G12D | ddPCR confirmed KRAS G12D. | Excision |
| TMF-152 |  |  | NA | Small biopsy |
| TMF-153 |  |  | NA | Small biopsy |
| TMF-154 |  |  | NA | Small biopsy |
| TMF-155 | KRAS_G12S | KRAS_G12S | Confirmed | Small biopsy |
| TMF-156 | EGFR_L858R | EGFR_L858R | Confirmed | Excision |
| TMF-157 |  |  | NA | Small biopsy |
| TMF-158 |  |  | NA | Small biopsy |
| TMF-159 |  |  | NA | Small biopsy |
| TMF-160 |  |  | NA | Small biopsy |
| TMF-161 | KRAS_G12C | KRAS_G12C | Confirmed | Small biopsy |
| TMF-162 |  |  | NA | Excision |
| TMF-163 |  |  | NA | Small biopsy |
| TMF-164 | KRAS_G12C | KRAS_G12C | Confirmed | Small biopsy |
| TMF-166 |  |  | NA | Small biopsy |
| TMF-167 | KRAS_G12C | KRAS_G12C | Confirmed | Excision |
| TMF-168 |  |  | NA | Small biopsy |
| TMF-169 | KRAS_G12C | KRAS_G12C | Confirmed | Small biopsy |
| TMF-170 |  |  | NA | Small biopsy |
| TMF-171 |  |  | NA | Excision |
| TMF-172 | EGFR_L858R | EGFR_L858R | Confirmed | Small biopsy |
| TMF-173 |  | BRAF_V600E | V600E confirmed by ddPCR | Excision |
| TMF-174 | KRAS_G12R | KRAS_G12R | Confirmed | Small biopsy |
| TMF-175 | KRAS_G12V | KRAS_G12V | Confirmed | Small biopsy |
| TMF-176 | EGFR T790M, S768I | EGFR_S768I , EGFR_T790M | Confirmed | Small biopsy |
| TMF-177 |  |  | NA | Small biopsy |
| TMF-178 |  | PIK3CA E545K | NA - PIK3CA not in OncoFOCUS | Small biopsy |
| TMF-179 | EGFR S768I, L858R | EGFR_L858R, EGFR_S768I | Confirmed | Small biopsy |
| TMF-181 |  |  | NA | Excision |
| TMF-182 |  | EGFR_L858R | L858R confirmed by ddPCR | Small biopsy |
| TMF-183 | EGFR N771_P772insR/H or P772_H773insT HP |  | NA - Mutation not in iPlexHS Panel | Small biopsy |
| TMF-184* | EGFR_p.E746-S752>V_c.2237-2255>T | EGFR_p.E746-S752>V_c.2237-2255>T | Confirmed | Excision |
| TMF-185* | EGFR D770_N771>AGG |  | NA - Mutation not in iPlexHS Panel | Small biopsy |
