# Supplementary material for: Assessment of common somatic mutations of EGFR, KRAS, BRAF, NRAS in pulmonary non-small cell carcinoma using iPLEX® HS, a new highly sensitive assay for the MassARRAY® System
Source: PLoS One. 2017 Sep 19;12(9):e0183715. doi: 10.1371/journal.pone.0183715 (PMC5604939; doi:10.1371/journal.pone.0183715)
Supplement: S2 Table — Table of mutants validated with ddPCR (Droplet Digital PCR System. Hercules, CA). Patient samples; TMF-28 (BRAF_V600E), TMF-37 (BRAF_V600E), TMF-63 (KRAS_G12C), TMF-69 (KRAS_G13D), TMF-80 (KRAS_G12D), TMF-104 (KRAS_G12V), TMF-144 (NRAS_G13R), TNF-173 (BRAF_V600E), TMF-182 (EGFR_L858R). Copy number and the microliter amount added to the PCR reaction. All samples were tested with a minimum of 4 biological replicates. Positive calls were the number of mutant calls out of the total number of biological replicates. Allelic frequency is presented as an average of the successful runs. Comments referring to “Present in OncoFocus spectrum but not significantly above baseline” is meant to indicate that there was a weak call which when present in the iPLEX® HS chemistry was sufficient evidence that the mutation was present in the old spectra. Please refer to Supplements S2 Table for the full list of patient samples orthogonally validated using ddPCR. (DOCX) [file pone.0183715.s002.docx]

**Supplemental Information**

| SampleID | Mutation | Copy # | ul/reaction | Total Copy # | Positive Calls | AF % |
| --- | --- | --- | --- | --- | --- | --- |
| TMF-28 | BRAF_V600E | 266 | 1 | 266 | 4/4 | 1.6% |
| TMF-37 | NRAS_G13R | 425.5 | 1 | 425.5 | 3/4 | 3.5% |
| TMF-63 | KRAS_G12C | 1200 | 1 | 1200 | 3/4 | 2.9% |
| TMF-69 | KRAS_G13D | 442 | 1 | 442 | 4/4 | 2.0% |
| TMF-80 | KRAS G12D | 2684 | 1 | 2684 | 3/4 | 3.9% |
| TMF-104 | KRAS_G12V | 848 | 1 | 848 | 4/4 | 3.0% |
| TMF-144 | NRAS_G13R | 466 | 1 | 466 | 4/5 | 3.7% |
| TMF-151 | KRAS_G12D | 1662.4 | 1 | 1662.4 | 4/4 | 8.0% |
| TMF-173 | BRAF_V600E | 1035 | 1 | 1035 | 4/4 | 1.3% |
| TMF-182 | EGFR_L858R | 240.4 | 1 | 240.4 | 4/4 | 3.8% |
