# Supplementary material for: Assessment of common somatic mutations of EGFR, KRAS, BRAF, NRAS in pulmonary non-small cell carcinoma using iPLEX® HS, a new highly sensitive assay for the MassARRAY® System
Source: PLoS One. 2017 Sep 19;12(9):e0183715. doi: 10.1371/journal.pone.0183715 (PMC5604939; doi:10.1371/journal.pone.0183715)
Supplement: S3 Table — Full list of all mutations covered in the iPLEX® HS panel. (DOCX) [file pone.0183715.s003.docx]

**Supplemental Information**

| **Gene** | **NCBI Ref Seq** | **CDS Mutation** | **AA Mutation** | **COSMIC ID** |
| --- | --- | --- | --- | --- |
| **BRAF** | NM_004333 | c.1406G>C | p.G469A | 460 |
|  | NM_004333 | c.1406G>T | p.G469V | 459 |
|  | NM_004333 | c.1781A>G | p.D594G | 467 |
|  | NM_004333 | c.1799T>A | p.V600E | 476 |
| **EGFR** | NM_005228 | c.2125G>A | p.E709K | 12988 |
|  | NM_005228 | c.2126A>C | p.E709A | 13427 |
|  | NM_005228 | c.2126A>G | p.E709G | 13009 |
|  | NM_005228 | c.2126A>T | p.E709V | 12371 |
|  | NM_005228 | c.2155G>T | p.G719C | 6253 |
|  | NM_005228 | c.2155G>A | p.G719S | 6252 |
|  | NM_005228 | c.2156G>C | p.G719A | 6239 |
|  | NM_005228 | c.2233_2247del(15)AAGGAATTAAGAGAA | p.K745_E749del | 26038 |
|  | NM_005228 | c.2235_2249del(15)GGAATTAAGAGAAGC | p.E746_A750del | 6223 |
|  | NM_005228 | c.2235_2248>AATTC | p.E746_A750>IP | 13550 |
|  | NM_005228 | c.2235_2251>AATTC | p.E746_T751>IP | 13552 |
|  | NM_005228 | c.2236_2250del(15)GAATTAAGAGAAGCA | p.E746_A750del | 6225 |
|  | NM_005228 | c.2236_2253del(18)GAATTAAGAGAAGCAACA | p.E746_T751del | 12728 |
|  | NM_005228 | c.2237_2251del(15)AATTAAGAGAAGCAA | p.E746_T751>A | 12678 |
|  | NM_005228 | c.2237_2254del(18)AATTAAGAGAAGCAACAT | p.E746_S752>A | 12367 |
|  | NM_005228 | c.2237_2252>T | p.E746_T751>V | 12386 |
|  | NM_005228 | c.2237_2253>TTGCT | p.E746_T751>VA | 12416 |
|  | NM_005228 | c.2237_2253>TTCCT | p.E746_T751>VP | 52935 |
|  | NM_005228 | c.2237_2257>TCT | p.E746_P753>VS | 18427 |
|  | NM_005228 | c.2237_2255>T | p.E746_S752>V | 12384 |
|  | NM_005228 | c.2238_2255del(18)ATTAAGAGAAGCAACATC | p.E746_S752>D | 6220 |
|  | NM_005228 | c.2238_2248>GC | p.L747_A750>P | 12422 |
|  | NM_005228 | c.2238_2252>GCA | p.L747_T751>Q | 12419 |
|  | NM_005228 | c.2239_2247del(9)TTAAGAGAA | p.L747_E749del | 6218 |
|  | NM_005228 | c.2239_2256del(18)TTAAGAGAAGCAACATCT | p.L747_S752del | 6255 |
|  | NM_005228 | c.2239_2256>CAA | p.L747_S752>Q | 12403 |
|  | NM_005228 | c.2239_2248TTAAGAGAAG>C | p.L747_A750>P | 12382 |
|  | NM_005228 | c.2239_2251TTAAGAGAAGCAA>C | p.L747_T751>P | 12383 |
|  | NM_005228 | c.2239_2258>CA | p.L747_P753>Q | 12387 |
|  | NM_005228 | c.2240_2251del(12)TAAGAGAAGCAA | p.L747_T751>S | 6210 |
|  | NM_005228 | c.2240_2254del(15)TAAGAGAAGCAACAT | p.L747_T751del | 12369 |
|  | NM_005228 | c.2240_2257del(18)TAAGAGAAGCAACATCTC | p.L747_P753>S | 12370 |
|  | NM_005228 | c.2303G>T | p.S768I | 6241 |
|  | NM_005228 | c.2307_2308ins(9)GCCAGCGTG | p.V769_D770insASV | 12376 |
|  | NM_005228 | c.2308_2309ins(9)CCAGCGTGG | p.V769_D770insASV | 12426 |
|  | NM_005228 | c.2309_2310AC>CCAGCGTGGAT | p.V769_D770insASV | 13558 |
|  | NM_005228 | c.2310_2311insGGT | p. D770-N771insG | 12378 |
|  | NM_005228 | c.2311_2312ins(9)GCGTGGACA | p.D770_N771insSVD | 13428 |
|  | NM_005228 | c.2319_2320ins(9)AACCCCCAC | p.H773_V774insNPH | 12381 |
|  | NM_005228 | c.2319_2320InsCAC | p. H773-V774insH | 12377 |
|  | NM_005228 | c.2369C>T | p.T790M | 6240 |
|  | NM_005228 | c.2389T>A | p.C797S | N/A |
|  | NM_005228 | c.2390G>C | p.C797S | 5945664 |
|  | NM_005228 | c.2573T>G | p.L858R | 6224 |
|  | NM_005228 | c.2582T>A | p.L861Q | 6212 |
|  | NM_005228 | c.2582T>G | p.L861R | 12374 |
| **ERBB2** |  | c.2324_2325ins(12)ATACGTGATGGC | p.A775_G776insYVMA | 20959 |
|  |  | c.2325_2326ins(12)TACGTGATGGCT | p.A775_G776insYVMA | 12558 |
|  |  | c.2326_2327ins(3)TGT | p.G776>VC | 12553 |
|  |  | c.2326_2327ins(3)TTT | p.G776>VC | 12552 |
| **KRAS** | NM_004985 | c.34G>A | p.G12S | 517 |
|  | NM_004985 | c.34G>C | p.G12R | 518 |
|  | NM_004985 | c.34G>T | p.G12C | 516 |
|  | NM_004985 | c.35G>A | p.G12D | 521 |
|  | NM_004985 | c.35G>C | p.G12A | 522 |
|  | NM_004985 | c.35G>T | p.G12V | 520 |
|  | NM_004985 | c.37G>T | p.G13C | 527 |
|  | NM_004985 | c.38G>A | p.G13D | 532 |
|  | NM_004985 | c.183A>C | p.Q61H | 554 |
|  | NM_004985 | c.183A>T | p.Q61H | 555 |
|  | NM_004985 | c.181C>A | p.Q61K | 549 |
|  | NM_004985 | c.181C>G | p.Q61E | 550 |
|  | NM_004985 | c.182A>C | p.Q61P | 551 |
|  | NM_004985 | c.182A>G | p.Q61R | 552 |
|  | NM_004985 | c.182A>T | p.Q61L | 553 |
|  | NM_006218 | c.1633G>A | p.E545K | 763 |
|  | NM_006218 | c.3140A>G | p.H1047R | 775 |
|  | NM_006218 | c.3140A>T | p.H1047L | 776 |
| **PIK3CA** | NM_006218 | c.1624G>A | p.E542K | 760 |
|  | NM_006218 | c.1633G>A | p.E545K | 763 |
|  | NM_006218 | c.3140A>G | p.H1047R | 775 |
|  | NM_006218 | c.3140A>T | p.H1047L | 776 |
