# Supplementary figures and images for: Assessment of common somatic mutations of EGFR, KRAS, BRAF, NRAS in pulmonary non-small cell carcinoma using iPLEX® HS, a new highly sensitive assay for the MassARRAY® System
Source: PLoS One. 2017 Sep 19;12(9):e0183715. doi: 10.1371/journal.pone.0183715 (PMC5604939; doi:10.1371/journal.pone.0183715)

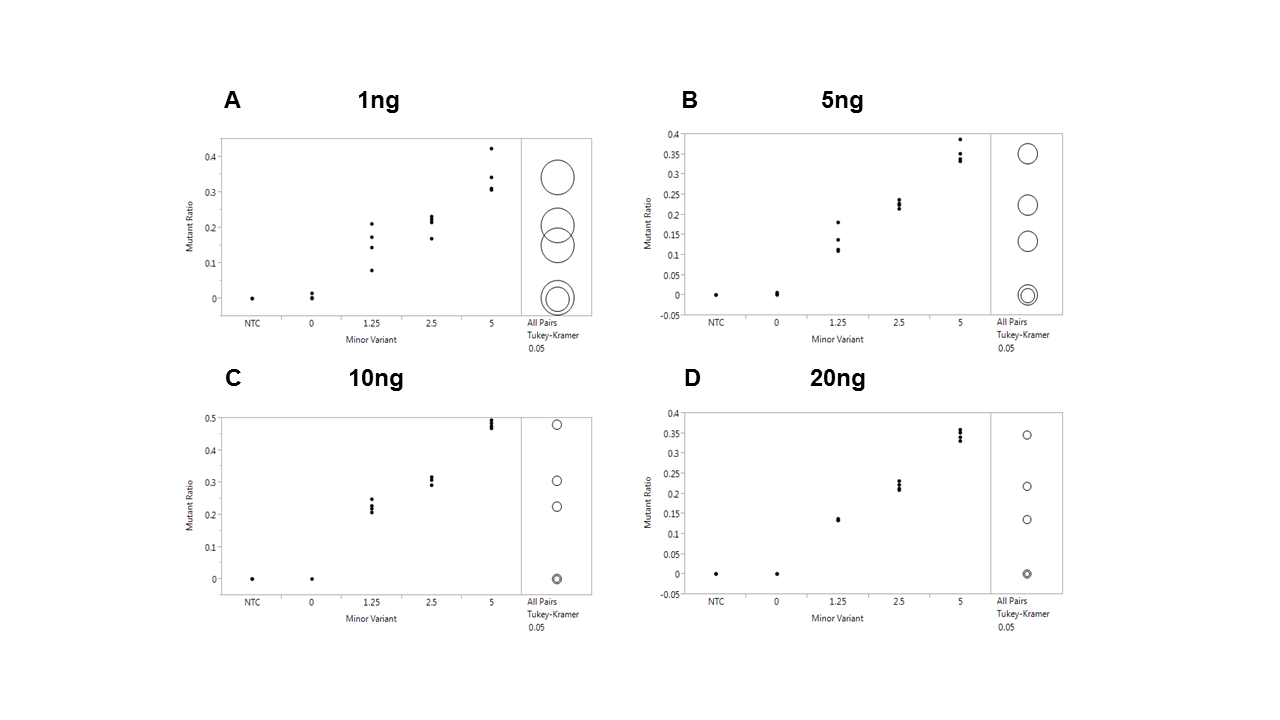

Supplement: S1 Fig — Mutation PI3KCA E542K was used a representative of all mutations tested. Graphs A-D are minor variant detection of PI3KCA E542K at input DNA concentrations ranging from 1ng, 5ng, 10ng and 20 ng. An all pairs Tukey-Kramer test was performed on all comparisons at a p value = 0.05. (TIF) [file pone.0183715.s004.tif]

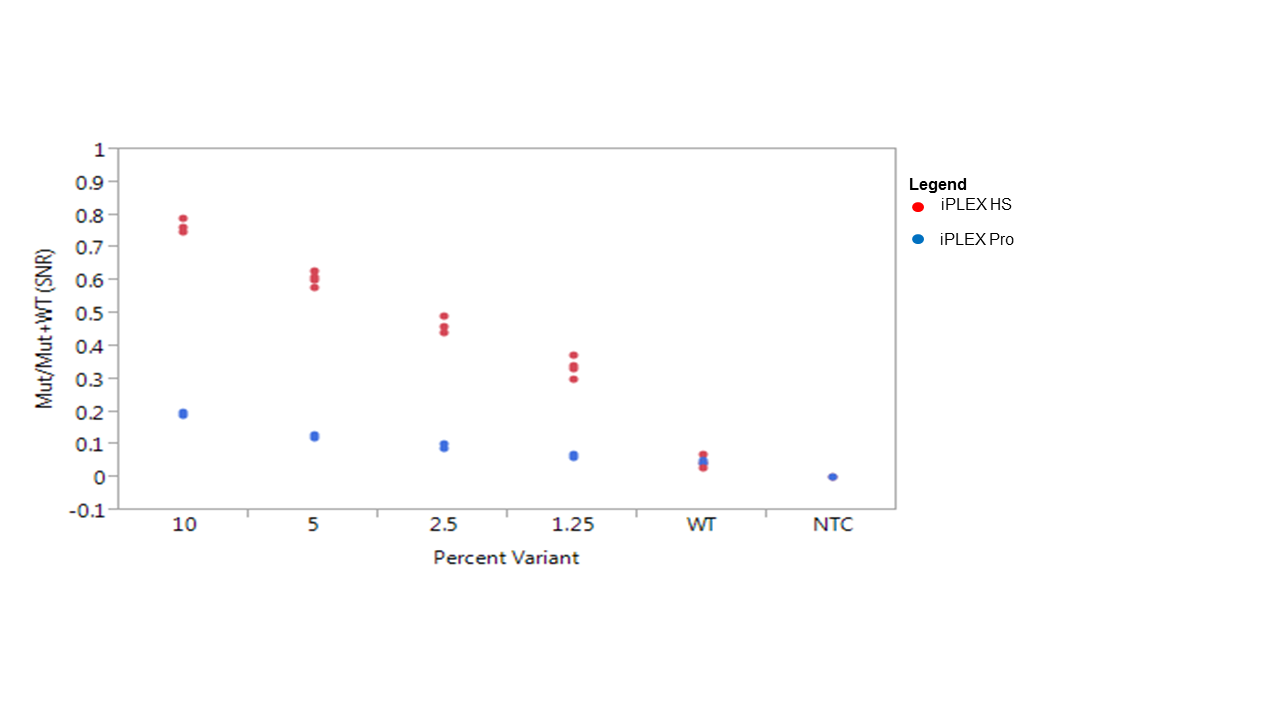

Supplement: S2 Fig — Comparison of iPLEX® Pro vs iPLEX® HS level of detection of minor variants. Data was analyzed using signal to noise ratio to identify prominent differences in peaks. (TIF) [file pone.0183715.s005.tif]
